# Estimating urban above ground biomass with multi-scale LiDAR

**DOI:** 10.1186/s13021-018-0098-0

**Published:** 2018-06-26

**Authors:** Phil Wilkes, Mathias Disney, Matheus Boni Vicari, Kim Calders, Andrew Burt

**Affiliations:** 1 Department of Geography, University College London, Gower Street, London, WC1E 6BT UK; 2 NERC National Centre for Earth Observation, Leicester, UK; 3 Earth Observation, Climate and Optical Group, National Physical Laboratory, Hampton Road, Teddington, TW11 0LW UK; 4 Computational & Applied Vegetation Ecology, Ghent University, Ghent, Belgium

**Keywords:** Above ground biomass, Urban forest, Airborne LiDAR, Terrestrial LiDAR, Allometry

## Abstract

**Background:**

Urban trees have long been valued for providing ecosystem services (mitigation of the “heat island” effect, suppression of air pollution, etc.); more recently the potential of urban forests to store significant above ground biomass (AGB) has also be recognised. However, urban areas pose particular challenges when assessing AGB due to plasticity of tree form, high species diversity as well as heterogeneous and complex land cover. Remote sensing, in particular light detection and ranging (LiDAR), provide a unique opportunity to assess urban AGB by directly measuring tree structure. In this study, terrestrial LiDAR measurements were used to derive new allometry for the London Borough of Camden, that incorporates the wide range of tree structures typical of an urban setting. Using a wall-to-wall airborne LiDAR dataset, individual trees were then identified across the Borough with a new individual tree detection (ITD) method. The new allometry was subsequently applied to the identified trees, generating a Borough-wide estimate of AGB.

**Results:**

Camden has an estimated median AGB density of 51.6 Mg ha^–1^ where maximum AGB density is found in pockets of woodland; terrestrial LiDAR-derived AGB estimates suggest these areas are comparable to temperate and tropical forest. Multiple linear regression of terrestrial LiDAR-derived maximum height and projected crown area explained 93% of variance in tree volume, highlighting the utility of these metrics to characterise diverse tree structure. Locally derived allometry provided accurate estimates of tree volume whereas a Borough-wide allometry tended to overestimate AGB in woodland areas. The new ITD method successfully identified individual trees; however, AGB was underestimated by ≤ 25% when compared to terrestrial LiDAR, owing to the inability of ITD to resolve crown overlap. A Monte Carlo uncertainty analysis identified assigning wood density values as the largest source of uncertainty when estimating AGB.

**Conclusion:**

Over the coming century global populations are predicted to become increasingly urbanised, leading to an unprecedented expansion of urban land cover. Urban areas will become more important as carbon sinks and effective tools to assess carbon densities in these areas are therefore required. Using multi-scale LiDAR presents an opportunity to achieve this, providing a spatially explicit map of urban forest structure and AGB.

## Background

Urban districts are often namesakes of the forests they have since replaced; in London for example, Norwood, Oakwood, Colliers Wood and Hainault were all once forests. Although the forest has long been cleared (some remnant individual trees may remain), urban landscapes still incorporate significant trees and areas of woodland as tree-lined streets, public and private gardens and parkland; collectively known as the urban forest. The ecosystem services provided by urban forests have long been recognised [1], for example, mitigating the urban “heat island” effect [2], providing habitat for city dwelling flora and fauna [3] and abating air pollution [4] (although see [5]) as well as aesthetic and well-being benefits [6]. These services have been valued at nearly $1 million km^2^ per annum [7] and individual urban trees can have a replacement value of up to £450,000 (~ $600,000) [8].

Another important ecosystem service provided by urban vegetation is the sequestration of carbon from the atmosphere. This is absorbed into plant tissue through photosynthesis and stored (sometimes for centuries) in woody tissues as biomass. Urban vegetation plays a disproportionate role in sequestrating anthropogenic carbon emissions as it is proximate to major sources i.e. vehicle emissions, as well as providing shade for buildings which reduce energy consumption [9, 10]. This biogenic sequestration of carbon by urban trees has been valued at £4.8 M ($6.3 M) per annum or £17.80 per tree in Greater London [10] and $2 bn per annum in the USA [11]. Large trees are of particular importance as they have the capacity to sequester more carbon than their smaller counterparts [9, 12]. Currently, however, the contribution of urban forests in the global carbon cycle is given little consideration, owing to their relatively small spatial area in terms of global forest cover [13]. Yet, as urban area is predicted to increase as a fraction of total land cover [14, 15], tools to accurately assess and monitor carbon stored in urban vegetation are required. Particularly as urban vegetation can be highly dynamic e.g. higher mortality [16] and faster growth rates [17] than natural forests, and methods designed for natural ecosystems may not be transferable to urban areas [18].

Above ground biomass (AGB) is defined as “the aboveground standing dry mass of live or dead matter from tree or shrub (woody) life forms, expressed as a mass per unit area” [19], typically Mg ha^–1^. Urban trees can account for up to 97% of urban AGB [20]. AGB can only be directly measured with destructive harvesting, an expensive and time-consuming approach that precludes remeasurement and is rarely practical beyond a handful of trees. For these reasons, AGB is often inferred through the use of allometric equations that associate more easily-measured parameters, such as diameter-at-breast-height *dbh* (usually measured at 1.3 m above the ground), tree height e.g. maximum crown height *H* or projected crown area *Ar*, with either stem volume *V* or AGB.

To scale up estimates of AGB beyond the tree level, inventory techniques are applied in both traditional forestry and urban studies [11, 20] where a representative sample of trees are measured. However, data acquisition for field inventory can be expensive, time-consuming and is often incomplete e.g. restricted to public lands; large area estimates then rely on scaling factors and land cover maps. Further, inventory data does not provide a spatially explicit map of the tree canopy and its attributes, which is useful for mapping other ecosystem services e.g. habitat extents, pollution dispersal etc.

Remote sensing presents an opportunity to capture synoptic, temporally frequent (every few days to weeks), fine spatial resolution data. This has already been widely applied to estimate AGB, across a range of scales, using both active and passive sensors from space based and aerial platforms [21–23]. In particular, light detection and ranging (LiDAR) techniques provide an unprecedented opportunity to capture high resolution, 3D information on tree and forest structure, such as canopy height, crown size and stem density [24, 25]. LiDAR instruments can be mounted on a range of platforms (hand held, tripods, vehicles, aeroplanes, satellites, etc.) that provide different scales information and detail. Two commonly referred to technologies are terrestrial and airborne laser scanning (aka TLS and ALS respectively); the former provides high fidelity information over a small spatial extents (10’s to 100’s of metres) whereas the latter offers synoptic data over large regional areas. Both TLS [26–28] and ALS [23, 29–31] have been used to estimate individual tree and stand level AGB.

Remote sensing methods for estimating AGB can be categorised into (i) area-based and (ii) individual tree detection (ITD) methods, where the latter are considered the state-of-the-art [30, 32]. Area-based methods use summary statistics of canopy structure to develop statistical associations with field inventory data, whereas ITD methods measure crown scale metrics to be used directly with allometry. LiDAR based ITD approaches can be grouped into two further categories dependent on data dimensionality; (i) image analysis of the rasterised canopy surface model (CSM), and (ii) cluster analysis of higher dimension datasets, typically $\mathbb {R}^3$ where the point cloud *xyz* coordinates are used. Image analysis often detect local maxima within the CSM, followed by expansion or watershed analysis to delineate crowns [16, 33].

Urban areas pose a particular challenge with regard to remote sensing of vegetation, where occlusion by tall buildings, high species diversity and heterogeneous and highly dynamic land cover add complexity to analysis. Tigges and Lakes [34] provide a review of the state-of-the-art of remote sensing to estimate urban AGB.

In urban areas, ITD has been achieved by combining ALS with hyperspectral imagery to identify trees [35], tree species [36, 37] and estimate leaf area index [38]. Regarding AGB, ITD has been applied to RapidEye [16] and Quickbird imagery [39] where crowns were subsequently attributed with LiDAR derived *H* to estimate AGB. Using a solely LiDAR based approach, Singh et al. [40] derived area-based AGB estimates from LiDAR predictor variables. Suggested advantages of a LiDAR derived ITD method to estimate AGB in urban area (as opposed to one from imagery) are (i) LiDAR data are more information rich [41] e.g. 3-dimensional and higher resolution (e.g. > 1 sample m^–2^), (ii) data is often acquired with greater overlap, including multiple viewing geometries, mitigating occlusion by tall buildings, and (iii) the 3D information inherent in LiDAR data can be used to segment trees based on their morphology as well as directly measure crown shape.

A common factor amongst the research discussed above is the use of high pulse density LiDAR data (e.g. > 10 pulses m^–2^), often acquired with complementary high resolution hyperspectral imagery, acquired over small spatial domains. Recently, government agencies and local authorities world-wide have opened their archives of spatial data, including ALS, under open data licence agreements. Harnessing this freely available resource could allow for large scale maps of urban vegetation attributes, such as AGB, to be computed without the cost of acquisition. Additionally, data is often acquired at regular temporal intervals that would allow for a Life Cycle Assessment of urban AGB [34]. However, a comprise of using these data is that it they are often captured for a different purpose e.g. flood-risk mapping, at a lower resolution and without coincident imagery. Therefore, newly developed techniques have to be adaptable and robust to differences in data quality.

As mentioned, allometric equations have long been used to estimate AGB, including in urban forests [9, 18]. However, the reliability of allometry (and it’s associated uncertainties) has been questioned owing to small, unrepresentative sample of destructively harvested trees or application outside the domain of observations (particularly diameter and mass) [42]. McHale et al. [18] compared allometry derived from trees grown in natural forest to that derived specifically for urban areas, noting large variability in AGB particularly at the tree scale. Vaz Monteiro et al. [43] computed allometry to estimate *H* and *Ar* from *dbh* for different UK cities; allometry for smaller trees were transferable between cities, whereas larger trees were prone to greater uncertainty. Further, understanding the range of allometric properties of urban trees, which tend to be grown under a wider range of pressures and constraints (water, space etc.) and display greater morphological plasticity (open-grown vs. closed canopy, management etc.), may help better understand the range of allometric variations in natural forests.

Recently, TLS methods have developed to accurately estimate the volume of individual trees; an approach known as quantitative structure modelling (QSM) [44, 45]. These methods have been shown to estimate tree AGB to within 10% of destructively harvested trees compared to up > 35% underestimation when applying species specific allometry [26, 27]. Further, as TLS is non-selective of trees captured, the allometry captures a range of structural conditions, including that of large trees. Lefsky and McHale [44] applied this approach to urban trees, reporting good agreement between QSM and field measured stem diameter.

## Methods

**Fig. 1:**
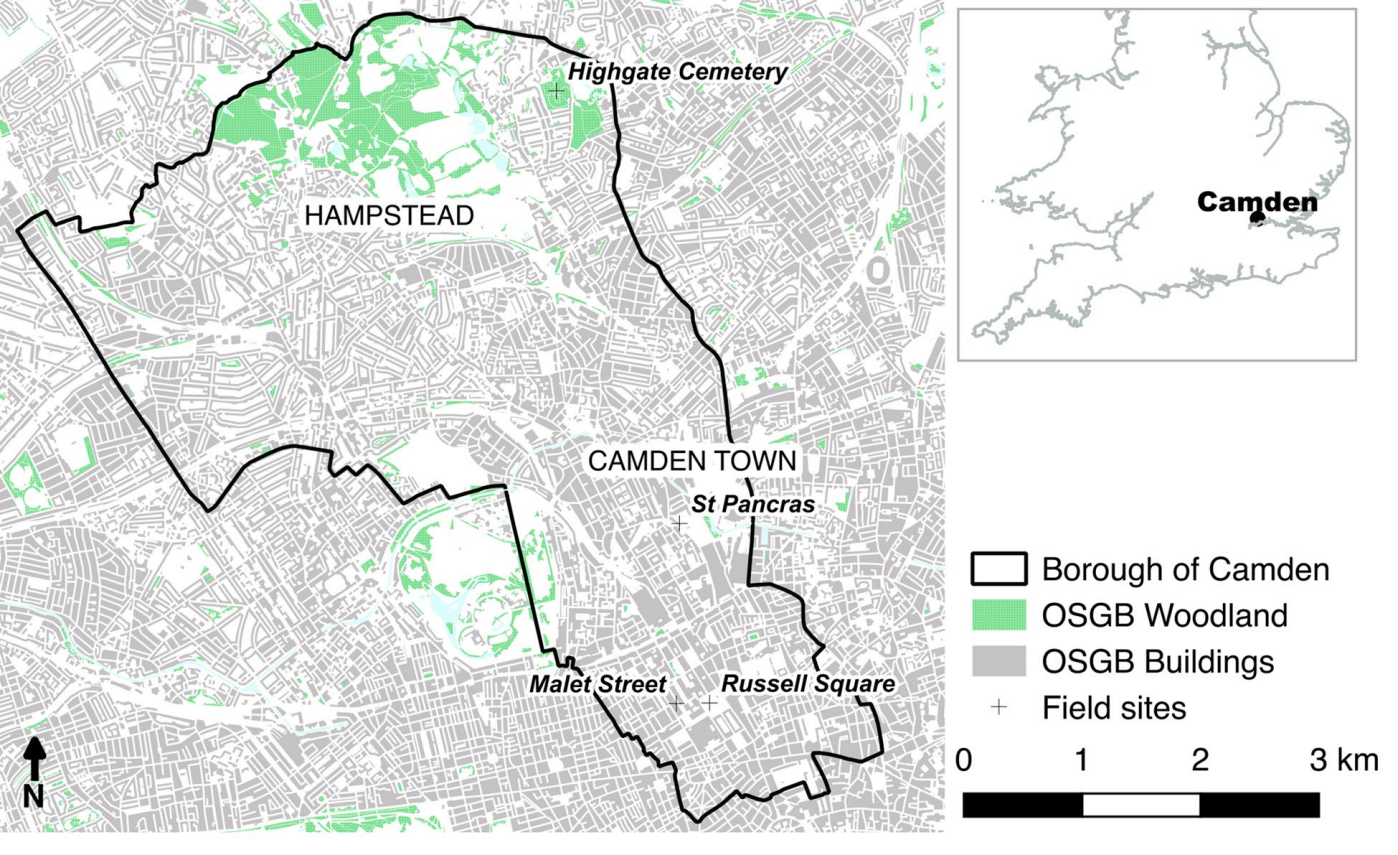
A map of the London Borough of Camden and location in UK (right). Field locations are identified in italics. Contains OS data ©Crown copyright and database right (2018)

Here we demonstrate a multi-scale LiDAR based approach to determine urban tree AGB for the London Borough of Camden, UK (Fig. 1). A new ALS ITD method is presented to identify and attribute individual trees with structure metrics. TLS is used to derive new allometry at four locations across the Borough, transferable tree structure metrics are identified and used to model tree volume. The new allometry is subsequently applied to the ALS segmented tree crowns to generate a Borough-wide map of AGB. To the best of our knowledge, LiDAR based ITD, to derive structural information for use in allometry, has not been previously applied in an urban context.

**Table 1:** TLS scanning location and description

Sites	Coordinates	Date	Leaf status	Type	Dominant species
Russell Square	51°31′18.0″N	8/2/2018	Off	Park	*P. acerifolia*
	0°07′33.5″W				
Malet Street	51°31′17.8″N	14/2/2018	Off	Street	*P. acerifolia*
	0°07′49.4″W				
St Pancras Old	51°32′08.4″N	18/7/2017	On	Churchyard and	*P. acerifolia*
Church and Road	0°07′50.5”W			Adjacent street	
Highgate Cemetery	51°34′06.3″N	10/8/2017	On	Urban forest	*F. excelsior*
	0°08′54.4″W				

### Location

The London Borough of Camden is located in inner north west London and comprises an area of 21.8 km^2^ (Fig. 1). The area was once forested but was extensively developed during the nineteenth and twentieth centuries to a mix of residential and industrial land use. Camden was chosen as it is typical of inner London Boroughs, containing a range of urban land cover types (“unmanaged” urban forest, large managed parks, tree-lined streets, private gardens, industrial areas and transport infrastructure e.g. train lines) encompassing a broad range of tree and forest management strategies, age structures, species composition and municipal functions. Camden also has good coverage of recent UK Environment Agency (UK EA) ALS. The Borough contains the suburbs of Camden Town and Hampstead, large areas of park land, including Hampstead Heath, and a number of smaller public squares and private gardens.

The Borough is home to ~ 28,000 street trees with an additional 10–15 K trees in parks and nature reserves [46]; however, this does not include trees located in City of London managed parks as well as other private land. For example, there are an estimated 30 K additional trees on Hampstead Heath in the north of the Borough (*pers. comm.* David Humphries, Trees Management Officer, City of London). Street tree species are dominated by *Platanus x acerifolia* (London Plane) 15% and *Tilia europaea* (Common Lime) 7%; all other species ($N=242$) comprise ≤ 4% each.

To derive new allometry for the Borough, four locations were scanned with TLS (Fig. 1 and Table 1). The locations were chosen for their representativeness of park and street trees in Camden, Highgate Cemetery was chosen after preliminary analysis suggested the area contained very high AGB.

### TLS acquisition and processing

TLS was captured with a RIEGL VZ-400 laser scanner (RIEGL Laser Measurement Systems GmbH) which has a beam divergence of 0.35 mrad, a pulse repetition rate of 300 KHz, a maximum range of 600 m and can record multiple returns. For all locations, the scanning resolution was set to an angular step of 0.04° as this has previously proved sufficient for tree extraction and QSM modelling [47]. As the RIEGL VZ-400 captures data in a panoramic field of view (100° in zenith when the scanner is upright), it is necessary to tilt the scanner by 90° to capture the full hemisphere. To capture data from multiple viewing positions and reduce the effects of occlusion, a number of scan positions were captured at each location (Table 2). To co-register scan positions it is necessary to have tie-points between scans that are easily identified in post-processing, here this was achieved using cylindrical retro-reflective targets mounted on poles [47]. Survey pattern was different for each location based upon tree density, leaf status, access and time constraints; mean distance between scan locations are presented in Table 2.

**Table 2:** Details of TLS scanning

Sites	Scan positions	Mean distance between positions (m)	Captured area (m^2^)
Russell Square	11	37	25,616
Malet Street	12	24	7786
St Pancras	19	24	25,392
Highgate Cemetery	25	13	4664

Area refers to the convex hull computed for the extracted trees

Point clouds from each scan were co-registered using RIEGL RiSCAN Pro software. Individual trees were then identified and extracted using the *treeseg* software library [48]. *V* was estimated using the QSM approach of Raumonen et al. [45], where the patch size variable $d_{min}$, which controls the size of cover sets used to generate cylinders (and ultimately the topological detail captured), was iterated over [48]. As the initialisation of each of QSM reconstruction is stochastic, 10 reconstructions for each tree point cloud and for each $d_{min}$ value were generated [26], this resulted in up to 160 reconstructions per tree. The set of reconstructions with the largest value of $d_{min}$ that produced satisfactory results [48] were chosen, from these the reconstruction with a volume closest to the mean was retained.

To reduce uncertainty in tree volume and subsequent allometry, point clouds and QSMs had to meet certain quality criteria to be considered for use in allometry development. These criteria were; (i) the mean nearest neighbour distance (computed as the mean Euclidean distance between a point and its four closest neighbours [47]) computed for each 1 m slice through a tree point cloud had to be ≤ 5 cm (excluding the uppermost slice), (ii) the 95% confidence level for the 10 QSM reconstructions for each tree point cloud had to be ≤  10% of volume, and (iii) the point cloud had to be unaffected by wind i.e. no shadowing of branches visible in the point cloud. The set of trees that fulfilled this criteria, referred to as QSM trees, were used to construct allometric equations (see below).

TLS extracted trees could not be reliably mapped to a tree species, instead a mean wood density value for the dominant species on a per location basis (Table 1) were taken from the *Global Wood Density Database* [49].

### ALS acquisition and processing

The UK EA capture ALS data over England primarily for flood risk mapping, this is distributed through an Open Government Licence by the UK Environment Agency as 1 km^2^ .las tiles [50]. Data for the area covering Camden were acquired on 6th February, 2015, at a pulse density of 2 pulses m^–2^ (calculated as the density of first returns in an open area) where for each outgoing pulse a maximum of 4 returns were recorded. Environment agency LiDAR data are captured to a vertical accuracy of ± 5 cm and a horizontal accuracy of ± 40 cm [51].

Data for the area intersecting the Camden Borough boundary were extracted from the global dataset. 5% of the Borough extent fell outside of the LiDAR footprint, previous UK EA acquisitions have been preprocessed to remove the majority of vegetation returns (Alastair Duncan, UK EA, *pers comm*) and were therefore unsuitable for filling gaps. Data were ground-normalised using the LAStools *lasheight* tool [52] so that *z* values were relative to the ground plane. A filter to remove points where $z \le 1$ m was then applied to remove ground and other low returns.

#### Segmenting trees from Airborne LiDAR

**Fig. 2:**
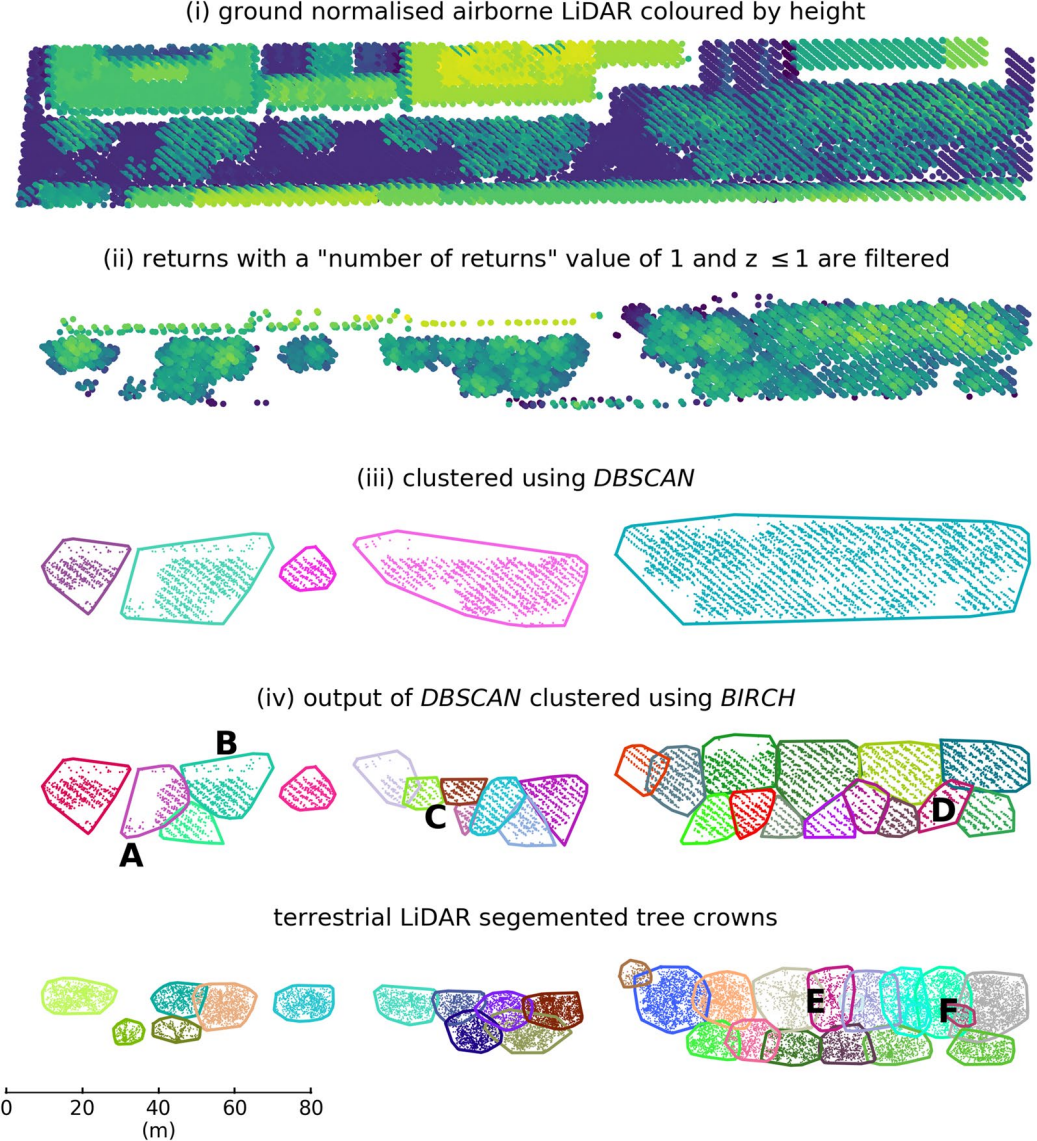
Individual tree detection work flow (i–vi) for segmenting ALS data into tree crowns, the bottom panel shows a TLS derived crown map as a comparison. Letters in panels 4 and 5 refer to common issues with the ITD crown segmentation where; **A** a small crown subsumed into a larger one, **B** remaining building points increasing crown area, **C** over segmentation of crowns, **D** commission errors, **E** under segmentation of crowns, and **F** omission errors (particularly of suppressed trees). Presented data is of Malet Street (Table 1)

Clustering techniques group individual data points into features sets that share some commonality. With regard to LiDAR data, features are often identified as groups of points connected in 3D space, such as street furniture [53] or tree crowns as discussed here. Some techniques require the number of features *a priori* e.g. *k*-means clustering, local maxima identified in the CSM are used to prime the algorithms as well as seed points from which clustering is initiated [29, 54]. Examples of cluster approaches that rely solely on the 3D point data included the *Mean Shift* algorithm [55] which uses a variable kernel to determine the search window size for which points are clustered and *PTrees* [56] which uses a multi-scale segmentation selecting the most likely segments as crown clusters. However, both of these approaches have only been applied to small forest plots and may not scale to large city-wide datasets owing to their complexity. Here we demonstrate a LiDAR point cloud based clustering approach that identifies individual tree crowns without additional imagery and that is scalable to large urban areas (Fig. 2).

A point cloud *D* contains points *p* where $D = \{p^N\}$ and $N = |D|$. Each $p \in D$ is a set of coordinates and other metadata associated with the .las format, for simplicity we need only consider $\{\mathbf {a}, rn\}$ where $\mathbf {a}$ = (*x*, *y*, *z*) coordinate vector and *rn* refers to the “Number of Returns” metafield [57]. The aim is to compute a set of clusters $C = \{c^N\}$ where cluster *c* corresponds to an individual tree crown. Each cluster $c =\{P, H, Ar, r\}$, where *P* is the point cloud that corresponds to the tree crown, *H* is the maximum $p_z \in P$, *Ar* is the projected crown area calculated as a 2D convex hull $\forall p \in P$ [58] and $r=\root \of {\dfrac{Ar}{\pi }}$, *r* was derived to simplify regression of crown dimensions with *H* (see below).

As urban areas are a patchwork of buildings, roads, trees, other green spaces etc., not all non-ground LiDAR returns are backscattered from tree crowns; therefore, $D = C + \epsilon$ where $\epsilon$ needs to be filtered before clustering can commence. This was achieved by firstly filtering *D* so that $\forall p \in D: p_{rn} > 1$ [59, 60]. This step removes the majority of buildings and other hard surfaces, which tend to backscatter a single return i.e. $p_{rn} = 1$ (Fig. 2ii). The majority of remaining points were resultant from vegetation backscatter, as well as from building edges, roof mounted air conditioning units and aerials, cranes etc [60]. This step also vastly reduces data volume, decreasing processing time in subsequent steps.

*D* was segmented into *C* using a two-step cluster approach. Here we use *Density-Based Spatial Clustering of Applications with Noise* (*DBSCAN*) [61] as a low pass filter to identify discrete tree crowns and canopies (Fig. 2iii) followed by *Balanced Iterative Reducing and Clustering using Hierarchies* (*BIRCH*) [62] to extract individual trees from canopy segments (Fig. 2iv). *DBSCAN* and *BIRCH* were both implemented using Python Scikit-Learn [63].

*DBSCAN* is suited to ITD from LiDAR point data as (i) |*C*| is not required as an *a priori* input, (ii) features can be of an arbitrary shape and size, (iii) outliers $\epsilon$ are removed, examples here include linear features e.g. building edges, where points do not fulfil the criteria (i.e. density) to form a cluster, and (iv) efficient scaling to large datasets. Ayrey et al. [64] used *DBSCAN* to identify and remove understorey shrubs from an ALS dataset captured over a conifer forest. *DBSCAN* requires two parameters, a neighbourhood radius *eps* and a minimum number of points *min_sample* so that *c* is considered a cluster when $|c_P| > min\_sample$ and $p \in c_P$ if $\Vert p - q\Vert < eps$. Values for *eps* and $min\_sample$ are a function of crown morphology and the ALS point density, $min\_sample$ increases monotonically with *eps*. If *eps* is too small, crowns tend to be split into sub-crown components (both horizontally and vertically) as well as an increase in false positive. If *eps* is too large then features of interest are ignored. Here, *eps* and $min\_sample$ were set to 3.5 m and 20 points respectively, this allows for smaller features to be identified ($\root \of {\pi 3.5}\approx 38$ m^2^) where point density ~ 2 points m^–2^.

**Fig. 3:**
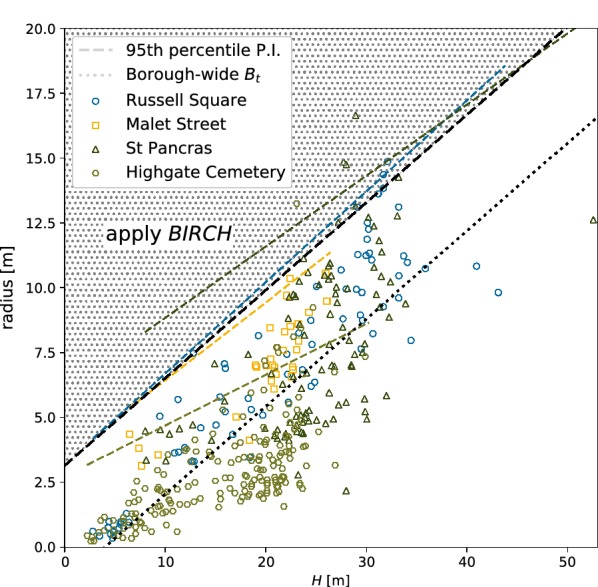
Local and Borough-wide thresholds for initiating *BIRCH* as well as the Borough-wide $B_t$ regression. Crowns that fall within the shaded area were further segmented with *BIRCH*

*DBSCAN* will concatenate adjacent, or *density-connected*, points into larger clusters that have a radius $>eps$ [61]. This is desirable as it allows *c* to have an arbitrary shape and size capturing the idiosyncrasies of a tree crown. However, this behaviour also leads to the merging of *c* into canopies, where points from adjacent crowns are in close enough proximity (Fig. 2). This is further exacerbated by low LiDAR point density that require lower values of $min\_sample$. *BIRCH* is therefore applied to further segment the output of *DBSCAN* into its constituent crowns if:

1 $$\begin{aligned} \beta + \alpha (c_{H}) < c_{r} \end{aligned}$$

where $\alpha$ and $\beta$ were determined empirically from a regression of TLS derived maximum canopy height with the 95${\mathrm {th}}$ percentile prediction interval of crown radius (Fig. 3). Prediction interval was chosen as the dependent variable to avoid segmenting larger crowns.

*BIRCH* is a hierarchical clustering algorithm that has two parameters; maximum radius of a cluster $B_t$ (if $c_r > B_t$ the cluster is split) and the total number of clusters $B_N$. $B_t$ was calculated in a similar way to the left hand side of Eq. 1 where instead crown radius was the dependent variable in the regression.

2 $$\begin{aligned} B_t = \beta + \alpha (c_{H}) \end{aligned}$$

Once *BIRCH* was initiated, it ran as a loop iteratively dividing *c* into smaller clusters for which $B_t$ was recalculated. Division of clusters ceased when $c_r \ge \beta + \alpha (c_H)$ for all new clusters. For each iteration of *BIRCH* was run twice; for the first run $B_N$ was not set allowing *BIRCH* to return a non-optimal set of clusters constrained only by $B_t$. For the second run $B_N$ is set to the number of crowns identified in the first iteration, this producing an optimal segmentation [63].

ALS ITD models were developed using the set of QSM trees from each location (‘local’) and using all QSM trees (‘Borough-wide’). For each model, the functions that were used to split large *c* and determine $B_t$ were computed as illustrated in Fig. 3.

#### Upscaling TLS volume estimates to ALS

Individual tree volume can not be directly measured with low pulse density ALS in a similar way to the TLS methods described above. Instead, ALS derived tree structure metrics are often used to infer volume and AGB. However, regression models computed using a suite of ALS variables can be idiosyncratic and only suitable for the domain in which they were derived [30]. In an urban context, there are a number of different forest types and scenarios which may preclude empirical modelling with multiple parameters. Further, as the aim is to extract and measure individual trees from both TLS and ALS instruments, metrics need to have an analogue for both measurement techniques. Considering these factors, maximum crown height *H* and projected crown area *Ar* were used as independent variables in the development of allometric equations [31, 33].

*C* was computed using the Borough-wide ALS model and exported as polygon vector layer of 2D crown envelopes attributed with *Ar* and *H*. Some cleaning was required ($<3\%$ of polygons) to remove duplicate trees (usually vertically offset) as well as false positives e.g building edges, cranes etc., these were easily identified as having maximum crown heights greater than expected. Polygons with an area < 10 m^2^ were also removed as the tended to coincide with building edges. TLS derived allometric equations were then applied to estimate *V* for each polygon. To convert *V* to AGB, an estimate of mean wood density was derived by mapping the trees in the Camden Council street tree database to a wood density value in the *Global Wood Density Database* [49]. Trees were first mapped at the species level ($N=9526$) and then, if no match was found, at the genus level ($N=10,973$); 287 trees could not be matched at either level and were disregarded. A mean wood density of 537 kg m^–3^ (*s*.*d*. 0.08 kg m^–3^) was used to convert *V* to AGB.

### Allometry uncertainty analysis

A Monte Carlo (MC) approach was used to identify and quantify uncertainties in allometry-derived AGB estimates [65, 66]. MC methods allow for complex and non-linear uncertainty to propagate to estimates of AGB. Estimates of uncertainty are computed by running the model *N* times where for each iteration the model input parameters are drawn from a probability density function (PDF) that characterises the uncertainty. Individual inputs can be also be isolated by freezing the other inputs, allowing for an estimate of their contribution to overall uncertainty.

Three potential sources of error were identified in the derivation and application of the allometry: (1) QSM estimates of *V*, (2) ALS-derived *H* and *Ar*, and (3) wood density values. Variability in TLS-derived tree structure parameters (*H* and *Ar*) were tested by random subsampling of TLS points clouds ($N=100,$ $\sigma =0.75$); RMSE for *H* was < 0.05 and < 1.8 m for *Ar*; therefore, TLS-derived structure was not considered in the MC analysis. QSM uncertainty was estimated on a per tree basis using the 10 reconstructions, the mean and standard deviation of *V* were used to parametrise a Gaussian PDF. A sample of $c \subset C$ ($N=250$) was used to estimate uncertainty in ALS derived crown structure. $c_P$ were randomly subsampled ($N=100$, $\sigma =0.75$) where *H* and *Ar* were calculated for each iteration. The standard deviation of *H* and *Ar* were then used to generate PDFs of measurement uncertainty for each extracted crown in *C*. Finally, a non-parametric PDF of wood density was constructed using wood density values mapped to each tree in the Camden street tree database.

For different scenarios, different sources of uncertainty were considered. When computing TLS AGB, wood density values were set to that of the dominant species, therefore, only QSM uncertainty was considered. When calculating ALS derived AGB at each of the TLS locations wood density was again assumed known and uncertainty in QSM and ALS measurements were computed. When computing AGB estimates for the entire Borough all sources of uncertainty were considered. For all scenarios, 100 MC simulations were run.

## Results

### TLS derived tree structure and AGB

A total of 385 trees were identified and extracted from the TLS data across the four locations. Of these, 99 trees (referred to as QSM trees) met the criteria for estimating tree volume (Table 3). A large number of trees were discarded from the QSM tree set for reasons including; (i) scanning domain did not cover the complete region of interest, therefore, trees on the periphery suffered from low point density, (ii) scan pattern were too sparse, particularly for St Pancras where leaf-on conditions resulted in high occlusion and low point density towards the top of the canopy and (iii) wind effects. Even light winds can produce “ghosting” in the point cloud which leads to an underestimation in stem volume, particularly towards the top of the canopy where poorly resolved branches are not identified in the QSM (see Fig. 11). Wind was not deemed to significantly impact *Ar*.

**Fig. 4:**
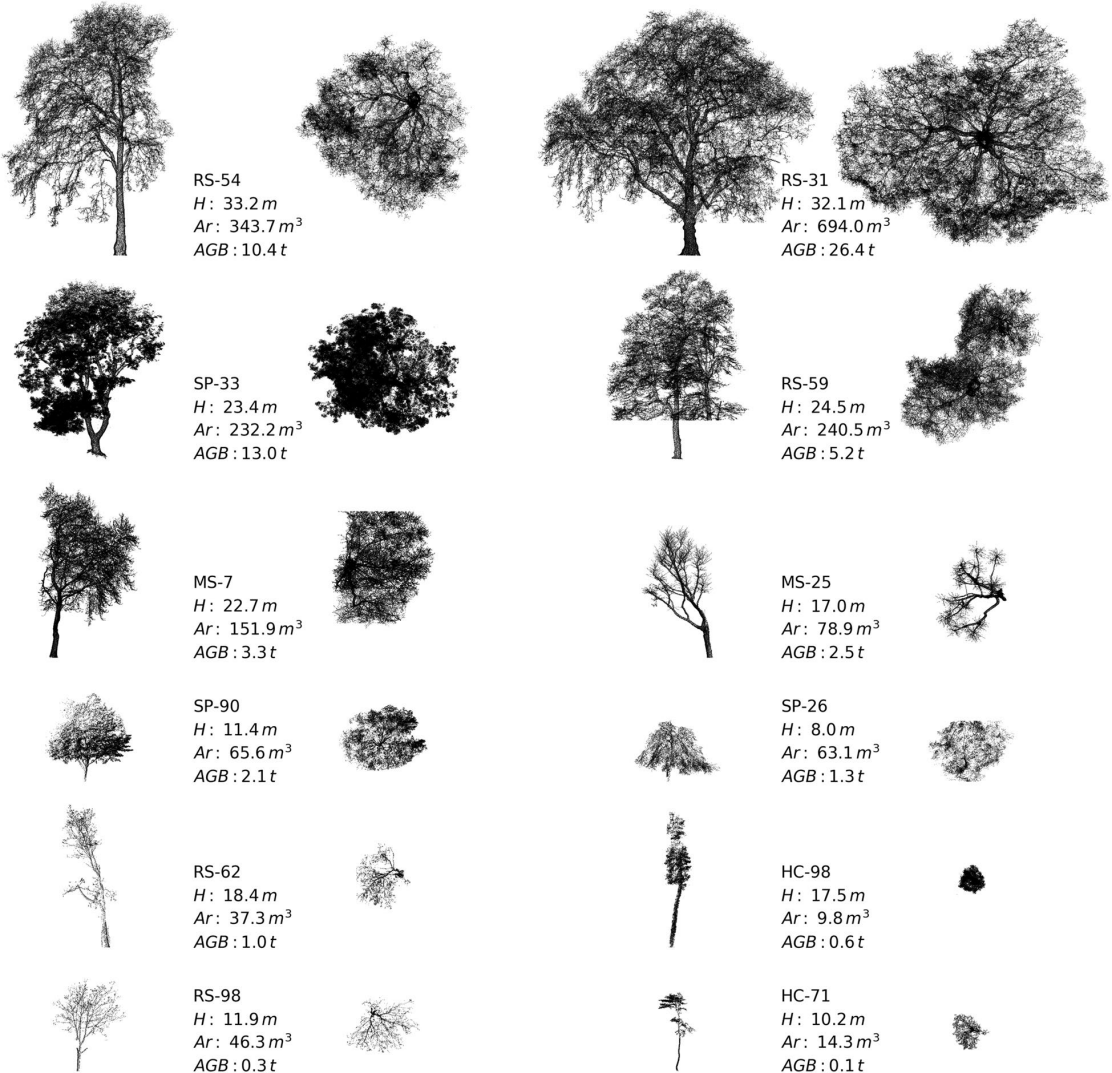
Profile (left) and plan (right) views of tree point clouds extracted from the TLS data. Tree codes refer to individual trees from Russell Square (RS), St. Pancras (SP), Malet Street (MS) and Highgate Cemetery (HS)

Of the QSM trees, the largest by height and volume were both *Platanus x acerifolia* located in Russell Square (RS-54 and RS-31 in Fig. 4 respectively). TLS measurements provided precise estimates of tree volume, particularly when captured in leaf-off conditions where 95% confidence level in QSM volume $\le 4\%$ (Table 3). Tree form is highly dependent on location and context e.g. trees that are found in street canyons have a strongly asymmetric crown shape (e.g. MS-25 and MS-7 in Fig. 4). Trees also vary in shape when grown in open parkland compared to those found in closed canopy forest, $\overline{Ar}$ is an order of magnitude smaller for closed-canopy forest trees (compare Highgate Cemetery and Russell Square trees in Fig. 4). Summary statistics of the extracted trees are presented in Table 3.

**Table 3:** Tree structure metrics and AGB estimates generated from TLS

Location	Trees			DBH		Tree height (m)		Projected crown area (m^2^)			Tree volume (m^3^)^a^		AGB (Mg ha^–1^)^c^	
	*N*	$\rho$ (ha$^{-1}$)	*QSM*	Mean	Max	Mean	Max	Mean	Max	$\sum$	Mean^b^	Max	Local	Borough-wide
Russell Square	78	30.4	25	0.78	1.65	20.6	34.1	214.2	694.0	16068	12.3 ± 4%	46.8	201.6 ± 2.2	193.3 ± 1.8
Malet Street	30	38.5	25	0.52	0.76	19.8	26.1	168.2	349.6	5047	6.4 ± 3%	14.3	124.8 ± 1.1	180.2 ± 2.9
St Pancras	97	38.2	9	0.60	1.55	24.7	33.9	214.4	871.9	20799	15.8 ± 7%	44.4	244.7 ± 10.5	258.8 ± 2.7
Highgate Cemetery	180	385.9	40	0.24	0.78	15.9	29.9	36.7	268.0	6604	1.2 ± 6%	8.0	276.3 ± 10.1	485.3 ± 7.6

As the volume of all TLS extracted trees could not be calculated confidently, AGB values are calculated using locally and Borough-wide derived allometry. It should be noted that AGB values are for inter-comparison purposes only and are not representative of likely AGB densities in a 1 ha area, for example, Malet Street is located in a highly developed area which is mostly devoid of trees

^a^ Tree volumes are for QSM trees only

^b^ 95% confidence level

^c^ *P. acerifolia* wood density value used except for Highgate Cemetery where *F. excelsior* was more common

**Fig. 5:**
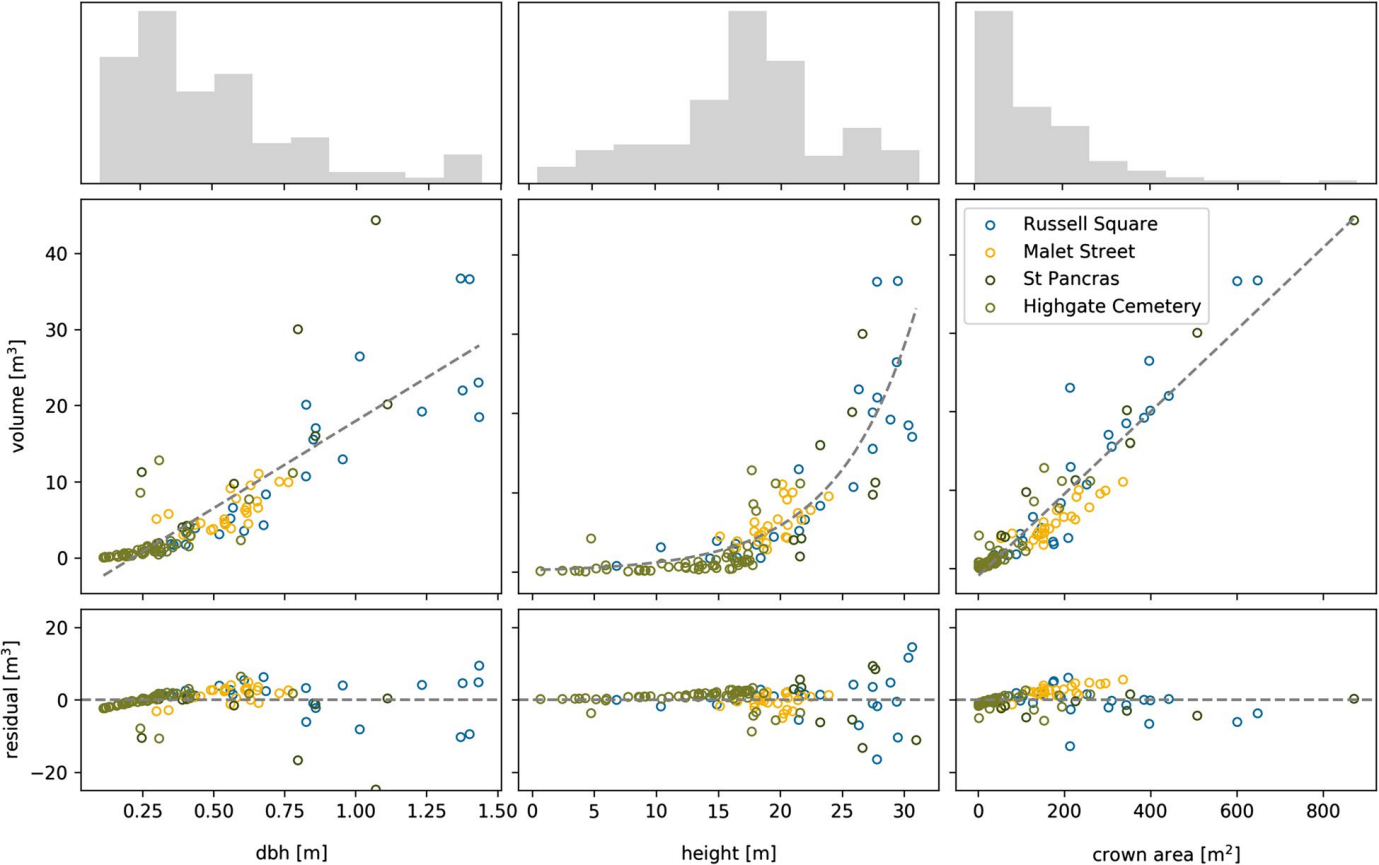
Regression between AGB and *dbh* (left), *H* (centre) and *Ar* (right). The top panel is combined frequency for all locations, the centre panel is regression of independent variable with *V* and the bottom panel are regression residuals

Allometry was derived using the set of QSM trees from each location (’local’) and all QSM trees (’Borough-wide’). Considering all QSM trees, *V* and *dbh*, *Ar* and $ab^H$ (where $ab^H$ is an exponential function, see Fig. 5) all showed $r^2>0.7$. A multiple linear regression was computed with *Ar* and $ab^H$ as independent variables ($p<0.001$) which explained 93.9% of variance in *V* (RMSE = 3.2 m^3^), the intercept was forced through the origin to avoid negative *V* for smaller trees. The allometric equation was subsequently applied to the polygon layer to estimate Borough-wide AGB. For the local allometry, $ab^H$ was not a statistically significant term ($p>0.01$).

**Fig. 6:**
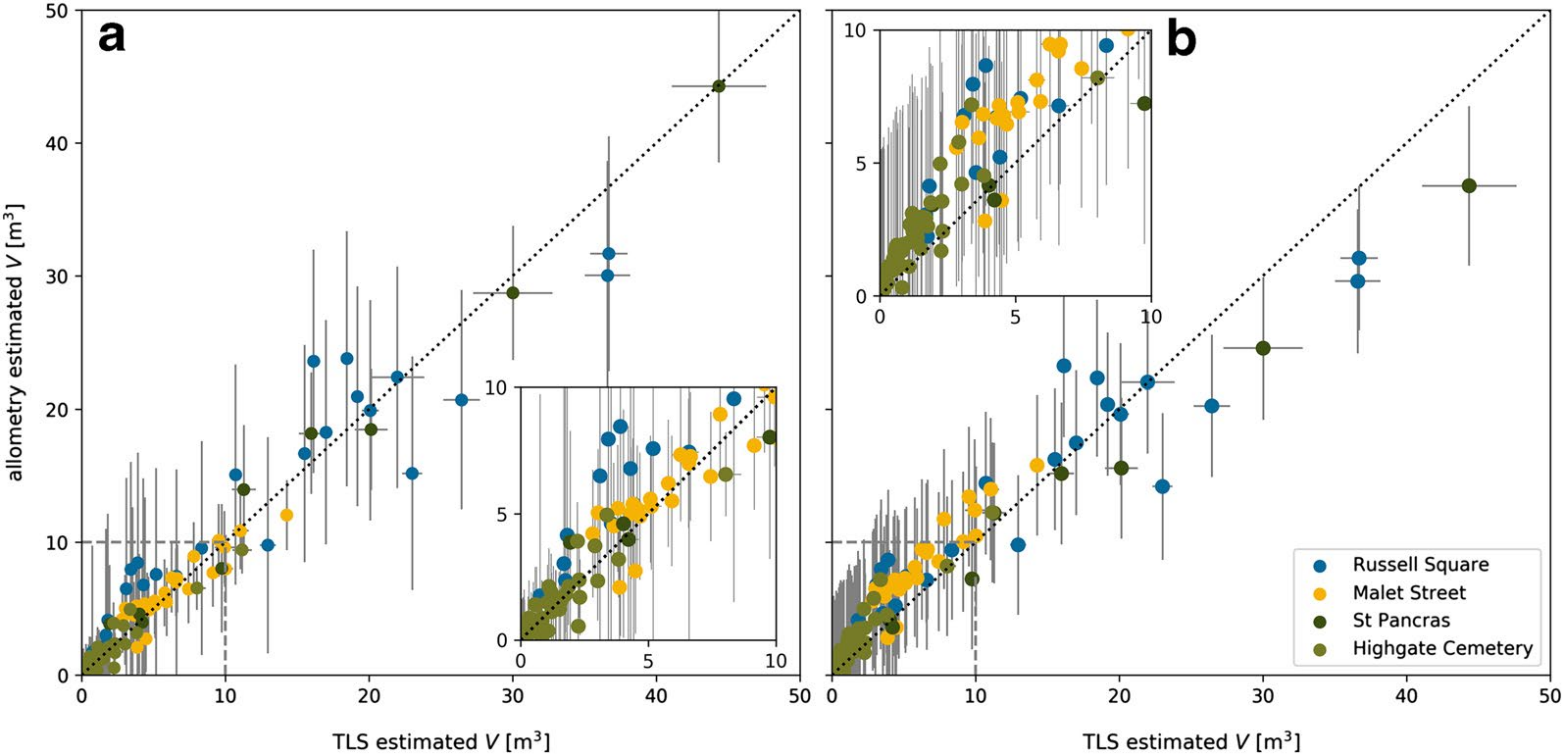
A comparison of QSM derived and allometry estimated *V* for the QSM trees. **a** Allometry was derived for each location (‘local’) and **b** using all QSM trees (‘Borough-wide’). Horizontal error bars represent the 95th percentile confidence level of tree volume from the 10× QSM model reconstructions and the vertical error bars represent prediction error from the regression. Inset panels magnify *V* between 0 and 10 m^3^

A comparison of TLS and allometry derived *V* (Fig. 6) shows that local allometry produced more accurate results than the Borough-wide equation (compare Malet Street trees in Fig. 6). The Borough-wide allometry tends to under and overestimate *V* of large trees ans small trees respectively. Large differences in allometry-derived AGB estimates are evident for Highgate Cemetery (Table 3) where the addition of *H* in the Borough-wide allometry significantly increases estimated AGB. This is due to the differing crown structure between open-grown and closed-canopy trees, where the former is dominant in the Borough-wide allometry i.e. open grown trees of a similar *H* have a much greater AGB. A comparison of trees with similar heights (e.g. MS-25 and HC-98 in Fig. 4) reveals that AGB for closed canopy trees can be a factor of ~ 5 less.

As all of the large trees (*H* > 10 m, $N = 26$) along Malet Street were successfully extracted from the TLS, a direct comparison of QSM computed and allometry estimated volume and AGB can be drawn. QSM derived AGB was 92.5 Mg, compared to local and Borough-wide derived allometry values of 93.8 Mg ± 1.1 Mg and 135.8 Mg ± 2.3 Mg respectively, suggesting allometry for this site overestimates AGB by 1.4 and 46.8% respectively. The overestimate of Malet Street *V* by the Borough-wide allometry can be seen in Fig. 6b. Applying allometry for *P. acerifolia* street trees from the US [67] estimates a growing stock volume of 80.5 m^3^ for Malet Street, compared to 165.6, 172.6 and 231.0 m^3^ for QSM, local and Borough-wide allometry; highlighting the requirement for caution when applying allometry derived for different circumstances.

### A comparison of TLS and ALS derived tree structure and AGB

**Fig. 7:**
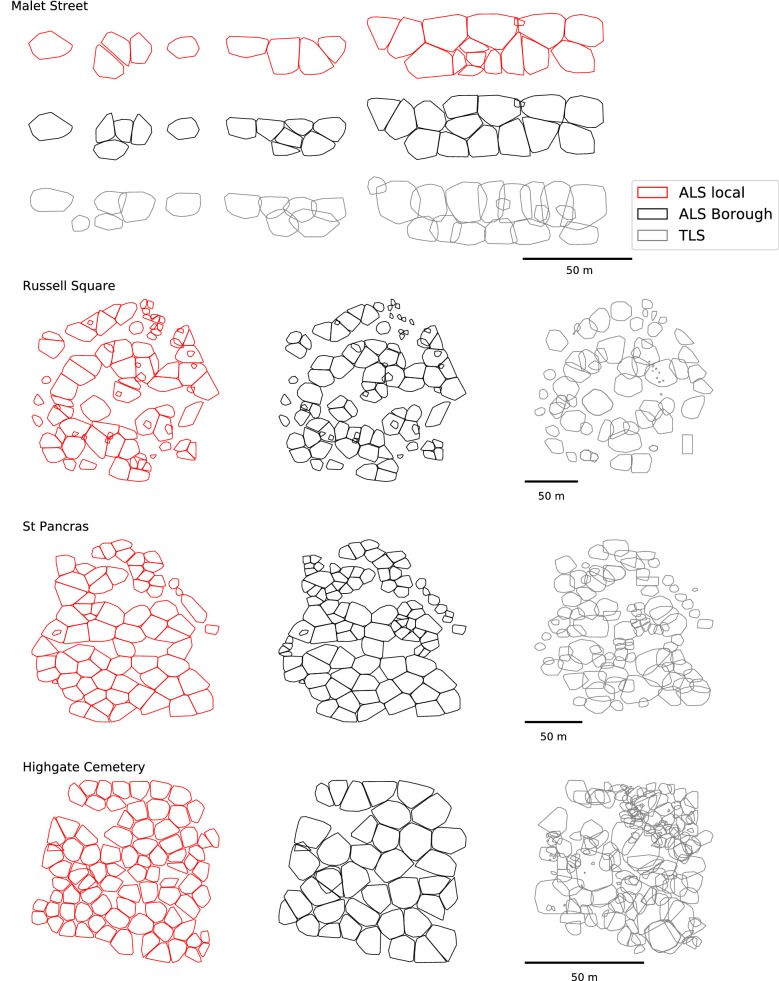
ALS derived tree crown polygons for local (red) and Borough-wide (black) ALS models, compared with TLS derived crowns (grey)

Summary statistics of ALS-derived crown metrics for each location are presented in Table 4 and a comparison of crown envelopes produced using TLS and local and Borough-wide ALS models is presented in Fig. 7. Both local and Borough-wide ALS models underestimate AGB by ≤ 25% compared TLS calculated values, where local parametrisation is slightly more accurate. The exception is Highgate Cemetery where AGB is underestimated by up to 55%. Both local and Borough-wide ALS models underestimate $\sum {Ar}$ as they are unable to resolve crown overlap (Fig. 7). When a model underestimates *N* trees, $\overline{Ar}$ is often overestimated to compensate and vice versa (Table 4).

**Table 4:** ALS derived crown structure and AGB estimates where *N* is number of crowns, $\overline{Z}$ is mean height, $\overline{Ar}$ is mean projected crown area, $\sum {Ar}$ is sum of projected crown area

Location	*N*	${\overline{Z}}$ (m)	${\sum _{Ar}}$ (m$^{2}$)	${\overline{Ar}}$ (m$^{2}$)	AGB (Mg ha^–1^)	
					Local	Borough-wide
Local						
Russell Square	88 (1.44)	17.2 (0.83)	15137.9 (0.95)	172.0 (0.70)	189.9 ± 12.1 (0.94)	164.7 ± 9.0 (0.85)
Malet Street	25 (0.96)	20.1 (1.02)	3905.1 (0.80)	156.2 (0.93)	96.6 ± 6.7 (0.81)	139.1 ± 8.1 (0.77)
St Pancras	70 (0.72)	22.6 (0.92)	18233.9 (0.92)	260.5 (1.21)	214.6 ± 13.3 (0.88)	204.3 ± 8.1 (0.79)
Highgate	84 (0.76)	15.8 (0.99)	3477.2 (0.60)	41.4 (1.13)	158.1 ± 45.7 (0.57)	238.5 ± 47.8 (0.49)
Borough-wide						
Russell Square	98 (1.61)	18.2 (0.88)	14495.7 (0.91)	147.9 (0.60)	181.9 ± 10.9 (0.90)	164.1 ± 9.7 (0.85)
Malet Street	28 (1.08)	20.3 (1.03)	3846.2 (0.79)	137.4 (0.82)	95.1 ± 7.7 (0.76)	140.0 ± 9.4 (0.78)
St Pancras	98 (1.01)	20.7 (0.84)	17721.4 (0.89)	180.8 (0.84)	208.5 ± 16.3 (0.85)	202.5 ± 10.5 (0.78)
Highgate	40 (0.36)	15.6 (0.98)	3809.2 (0.66)	95.2 (2.59)	164.9 ± 15.9 (0.60)	219.1 ± 18.8 (0.45)

Values in parentheses indicate the fraction compared to TLS estimated values where > 1 suggests an ALS overestimate and vice versa

Different parameters sets (derived locally or Borough-wide) were used when segmenting crowns (see Eqs. 1, 2 and Fig. 3)

At Highgate Cemetery, forest structure is not characterised well with either the local or Borough-wide ALS models. For example, *N* trees is underestimated by 14 and 64% respectively compared to the TLS estimate and *Ar* coefficient of variation is ~ 32% for both ALS models, compared to 100% for TLS-derived *Ar*. Differences between ALS and TLS identified crowns are caused by an uneven age structure of a mix of older trees with large crowns and younger trees filling canopy gaps (Fig. 7). All trees have similar *H* however, therefore, *BIRCH* will compute a similar crown radius during segmentation (Eq. 2). Other suggested reasons for poor characterisation include low ALS pulse density not characterising individual crown morphology and a relatively small capture area that compounds scaling errors.

### Borough wide estimate of AGB

**Fig. 8:**
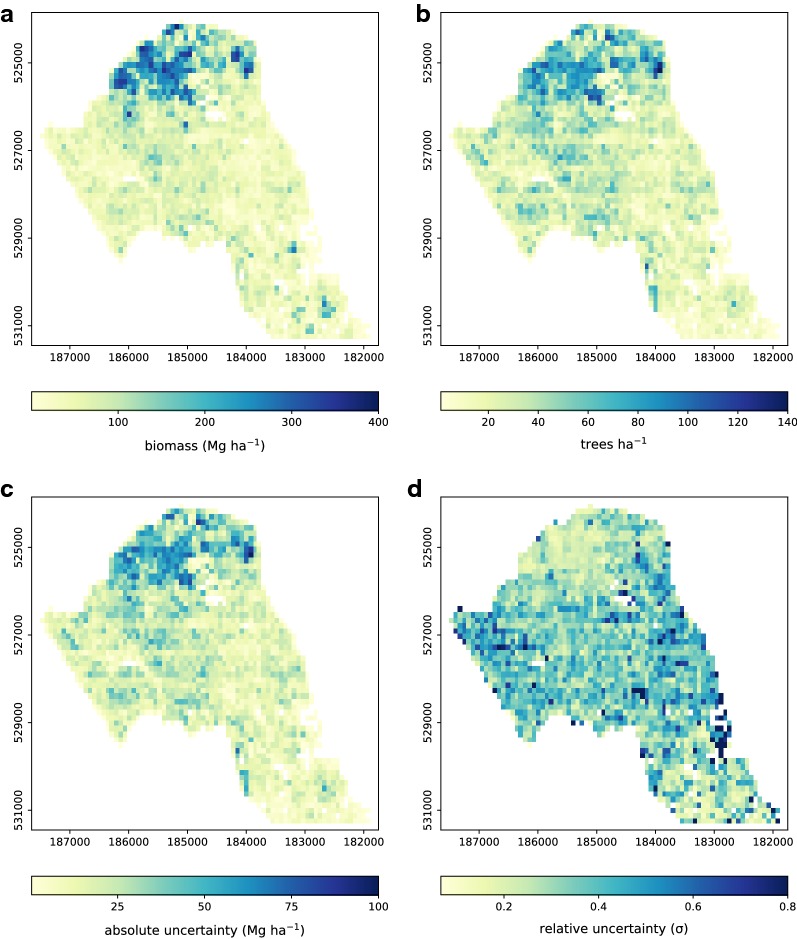
Borough-wide maps of ALS derived AGB density (**a**), tree density (**b**) and absolute (**c**) and relative uncertainty (**d**)

Camden has an estimated median AGB density of 51.7 Mg ha^–1^ (*s*.*d*. 68.5 Mg ha^–1^) and a maximum density of 376.5 Mg ha^–1^ situated in the Hampstead Heath area (Fig. 8). Maximum values are likely to be an overestimate owing to the poor representation in the allometry as discussed previously. A total of 84,282 individual tree crowns were identified across the Borough, median and maximum tree densities were 36 and 215 trees ha^–1^ respectively. High AGB areas are concentrated to the north of the Borough (Fig. 8) and are coincident with areas of maximum tree density. ALS-derived tree density values for the forested areas is likely to be an underestimate as TLS estimates for tree count in Highgate Cemetery are 385 trees ha^–1^ (Tables 3 and 4).

**Fig. 9:**
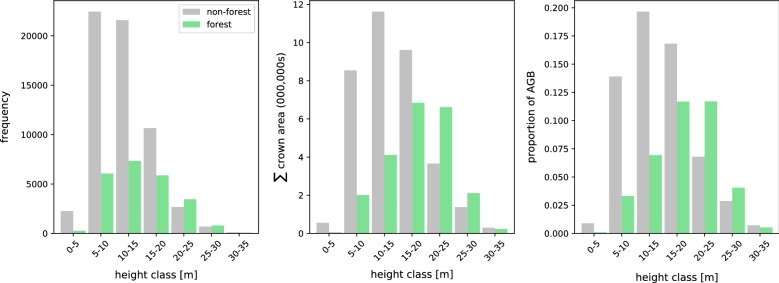
Histograms of tree count (left), sum of crown area (centre) and proportion of AGB (right) as a function of tree height class. Trees have been classified into forest and non-forest using the OSGB forest extent map (see Fig. 1)

Trees in non-forest areas where $10< H < 15$ m account for ≥ 25% of trees and ~ 20% of total AGB (Fig. 9). Trees in forested areas account for 38% of total AGB where forested areas account for $<8\%$ of total land cover. Large trees i.e. trees where *H* $\ge$ 30 m, account for < 2% of total AGB, these large trees are more common in non-forest areas in the south of the Borough. The tallest and largest volume trees identified in the ALS were 36.0 m and 35.0 m^3^ respectively, both were located in *Gray’s Inn Fields*.

Uncertainty in AGB can be > 100 Mg ha^–1^ (95% confidence level); however, greatest uncertainty as a proportion of AGB occurs in areas of low AGB (Fig. 8). MC simulations indicate AGB is estimated to ± 30%, the largest source of uncertainty is wood density which accounts for ~ 65% of overall uncertainty. ALS measurement uncertainty and QSM volume uncertainty account for 30 and 5% respectively.

## Discussion

### Urban areas as a carbon sink

To inter-compare carbon (C) densities with other cities and ecotones, AGB values are converted to C by multiplying by a factor of 0.471 [68]. Median carbon density for Camden is 24.3 Mg C ha^–1^, this is significantly higher than previously published estimates for inner (16.1 Mg C ha^–1^) and Greater London (14.8 Mg C ha^–1^) [10]. The distribution of AGB is likely skewed to the right by an overestimate of “forest” C density calculated with the Borough-wide allometry (Table 3), although Camden does have a greater proportion of park land compared to inner London [69]. For non-forest areas, median C density is 18.9 Mg C ha^–1^ which is again higher than reported inner London values. The ALS predicted number of trees is much less than the mean value previously reported for London (51 tree ha^–1^) [10] and the mean value for UK towns (58.4 tree ha^–1^) [1]; reasons for this include smaller trees being either subsumed into or occluded by larger trees using ALS ITD, whereas the i-Tree Eco and other protocols record all trees where *dbh* >7 cm [1, 10].

Compared to other UK towns, Leicester has a much higher C density (31.6 Mg ha^–1^) [20] whereas Edinburgh (16 Mg C ha^–1^) [70] and Torbay (15.4 Mg C ha^–1^ [69] are considerably lower. A comparison with other European cities suggests that Camden has a much higher biomass density, for example, Barcelona [71] and Berlin [34] have mean C densities of Berlin 7.3 and 11.2 Mg ha^–1^ respectively. Lower densities for Berlin could be due to smaller mean tree size where mean tree mass is 372 kg compared to 882 kg in Camden. A comparison with cities globally; major cities in the US have a mean C density of of 7.7 Mg C ha^–1^ [72] and major Chinese cities have a mean of 21.3 Mg C ha^–1^ [73].

Considering “woodland” areas, using the locally calibrated TLS data, estimated C density for Highgate Cemetery is 132.4 Mg C ha^–1^. This compares to Leicester which has a C density of 280.6 Mg C ha^–1^ for mixed ownership woodland and 287.6 Mg C ha^–1^ for public ownership [20] which are considerably higher. UK forest and woodlands have a mean density of 53.6 Mg C ha^–1^ [74]; therefore, forested areas of Camden could be considered AGB “hotspots”. In the US, the forests surrounding Seattle have a density of 104 Mg C ha^–1^ for mixed forest and 166 Mg C ha^–1^ for conifer forest [75]. US forests have a mean density of 53.5 Mg C ha^–1^ [76].

**Fig. 10:**
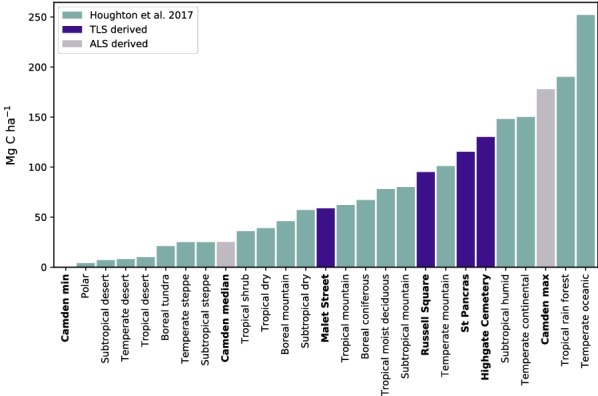
A comparison of median C density for different ecotones [92] with TLS and ALS derived values for Camden. AGB was converted to C using a conversion factor of 0.471 [68]

A comparison with C sinks from different ecotones is presented in Fig. 10. This shows that, although the contribution of urban areas to global AGB maybe relatively small owing to the limited spatial extent, some urban forests have AGB density comparable to tropical and temperate forests. Therefore the importance of conserving these areas as AGB sinks can not be understated, particularly locally.

It should be noted that values presented above were computed using very different data processing and analysis methods which may hinder inter comparison [41]. For example, techniques vary from using ALS (this study), interpretation of satellite imagery [16] or aerial photos [77], field inventory where plots are located per land class [20] or along transects [75]. As a comparison, mean C density for Leicester is estimated as 31.6 Mg ha^–1^ using a stratified sample of inventory plots in conjunction with published allometry [20]. Applying the method presented here to 2014 UK EA ALS data captured for the same area (and using the Borough-wide allometry) computes a much lower C density of 9.1 Mg ha^–1^.

### Using TLS to estimate AGB and derive allometry

This study highlights the importance of applying allometric equations in the correct context and with prior knowledge of their derivation. For example, a difference of >200 Mg ha^–1^ was computed at Highgate Cemetery by applying location specific and Borough-wide (yet still local) allometric equations. A large difference in total *V* was also noted when applying an equation from the literature [67], compared with local and Borough-wide allometry for Malet Street. Computing locally applicable allometric equations is not always feasible, however, as demonstrated by Calders et al. [26] and Gonzalez de Tanago Menaca et al. [27], as well as here, TLS measurement can be used to derive unbiased allometry quickly and non-destructively.

**Fig. 11:**
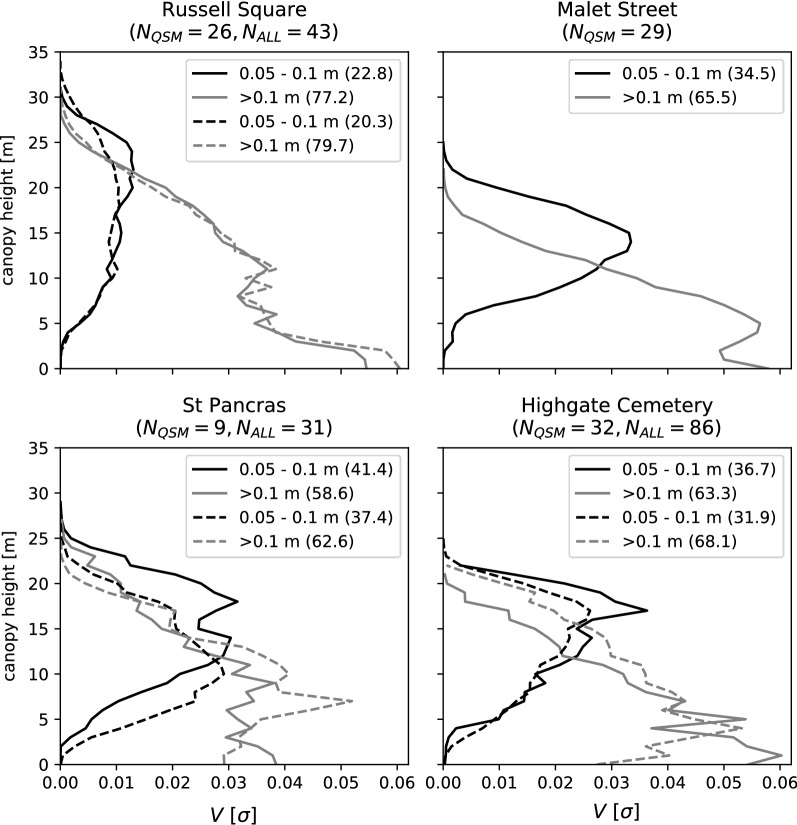
Vertical profiles of QSM derived tree volume classified into small (0.05–0.1 m diameter) and large (> 0.1 m) branches. Solid lines ($N_{QSM}$) are produced using QSM trees only, dashed lines ($N_{ALL}$) are for all QSM models (regardless of quality). Number in parentheses are the percentage of total AGB. Branches with a diameter of < 0.05 m were removed from analysis

Widely applied allometric equations (e.g. Chave et al. [78]) often include a *dbh* term, due in part to theoretical scaling laws of tree mass [79] as well as ease of measurement. From an airborne or satellite remote sensing perspective, *dbh* can only be inferred and is therefore modelled as a function of other variables such as *H* and *Ar* [31]. As demonstrated here, a linear combination of $ab^H$ and *Ar* explained 93.9% variance in *V* and was therefore suitable for deriving new allometry that excludes a *dbh* term. Others have also omitted a *dhb* term, using *H* and *Ar* to estimate *V* and AGB from airborne LiDAR [33, 66]. In fact, both $ab^H$ and *Ar* explained more variance than *dbh* for the QSM trees; however, this may be unique to urban trees where tree management e.g. pollarding, may cause deviation from a theoretical ideal. The strong linear association between *V* and *Ar* can be explained by the relativity high proportion of *V* distributed in the tree crown (Fig. 11), particularly for small diameter branches (ø ≤ 20 cm) which can constitute 20–40 % of AGB. Goodman et al. [80] noted a similar trend for trees in tropical forests.

Using the Borough-wide allometry, RMSE for predicted tree level AGB was 1.8 Mg where model residuals show a degree of heteroskedasticity. This is likely due to plasticity in crown shape caused by location (open park land, closed canopy forest, street canyon) as well as factors of competition for space and resources (artificial watering), pollution exposure, management strategies etc. Vaz Monteiro et al. [43] conclude that applying allometry to large trees grown in different locations across the UK results in significant uncertainties. Here, however, error (as a proportion of tree volume) is more evident in smaller trees (AGB < 10 Mg). This is due to taller QSM trees having similar characteristics (open-grown) whereas there a larger number of small trees with a high degree of variability in tree structure.

To convert *V* to AGB requires an estimate of wood density, this represented the largest uncertainty when estimating AGB. Here a mean value was applied to all trees derived from the Camden street tree database. However, in Highgate Cemetery (and most likely other wooded areas) the most common species were *Fraxinus excelsior*, fortunately this has a similar wood density to the mean of 560 kg m^–3^ [49]. Fusion of LiDAR and spectral data may allow for more accurate identification of tree species and from which to derive wood density values [34, 37].

### Airborne LiDAR to estimate tree volume

Considering ITD methods, applicability of either cluster analysis or CSM based methods is likely to be forest type (e.g. tree density) and sensor/data dependent [30, 81–84]. Currently is dense tropical forests, a CHM approach proved more reliable [30]. However, cluster analysis are increasing in popularity owing to new techniques, increased computing power and instrument capability [48]. A cluster approach was developed here that utilises the unique characteristics of trees when scanned with LiDAR, such as multiple interceptions of LiDAR pulses and predictable tree morphology.

An advantage of *DBSCAN* is that it is responsive to tree morphology without *a priori* information of canopy structure. *BIRCH*, on the other hand, segments larger canopy clusters into crowns of similar sizes where *H* is similar regardless of underlying morphology, this caused errors in the representation of crown structure e.g. Highgate Cemetery (Fig. 7). If higher pulse density ALS was available, the *BIRCH* step could possibly be replaced by a CSM watershed based approach to identify crown extents from canopy clusters. Regardless, it is suggested that future urban studies first discard points where $p_{rn} = 1$ to facilitate the identification of vegetation.

When compared to TLS estimated canopy and crown structure, ALS tended to underestimate crown height and projected crown area (Table 4). Underestimation of *H* is a common error associated with ALS as pulses often miss the apex of the tree [24], an issue exacerbated by low pulse density. Underestimation of crown area is caused by ALS not being able to delineate overlapping crowns satisfactorily (Fig. 7). Increased crown overlap is common in urban areas owing to tree management practices e.g. closer tree spacing than naturally occurring, reduced resource competition, pollarding etc. Tigges et al. [16] reported an underestimate of tree numbers (~20%) when applying ITD to Rapideye captured over Berlin. Our approach was more accurate for street and park trees (Table 4) as smaller (i.e. *Ar* < 100 m^2^) and sub-dominant trees were identified [aided by a winter (leaf-off) ALS capture]. In “forest” areas ALS ITD performed less well, underestimating the number of trees and overestimating their mass. Overestimated mass was caused by under-representation of closed-canopy forest in the Borough-wide allometry. Applying a land-cover classification and computing land-cover specific allometry may reduce errors in AGB estimates; however, errors may be exacerbated by poor classification or land cover definitions.

The ALS ITD method satisfactorily identified and attributed individual trees, despite the relatively low pulse density of the data. Maps of individual tree structure are not only useful for estimating AGB, but could also be applied to pollution dispersion [85] and habit extent modelling, for example. The utility of open-access, large area LiDAR datasets is yet to be fully realised for vegetation mapping, particularly LiDAR in urban areas. In England for example, 70% of the land area is covered by airborne LiDAR data (although see earlier comments regarding processing level) with multi-temporal coverage available for certain areas. Recent advances in LiDAR technology, such as the ability to record full waveform backscatter, has also allowed for more accurate mapping of urban vegetation i.e. identifying understorey and suppressed trees [86, 87]. However, full-waveform LiDAR capture at a city wide scale is still experimental, expensive to capture and store and complex to analyse [87]. Alternatively, data fusion of passive (e.g. multi- and hyperspectral sensors) and active sensors (including mobile scanners [88]), as well as inclusion of open source or freely available data (e.g. Google Street View [89, 90]) could be used. Multiple data streams could create a temporally rich analysis that allows for an urban AGB Life Cycle Assessment [34] as well as for application in protocols (i.e. i-Tree Eco protocol [91]) which combine meteorological data with tree structure metrics to determine a suite of ecosystem services.

## Conclusions

Increasingly, urban trees are being valued for all the ecosystem services they can provide, including as an AGB sink. Although urban areas are currently a small proportion of total land cover, urbanisation is predicted to increase long into the century; therefore, an effective tool set to measure urban AGB, as well as other tree structure metrics, is required. Advances in remote sensing technology are allowing for new methods to more accurately map forest AGB. In particular, LiDAR technologies, both terrestrial and airborne, allow for highly detailed information on tree structure to be derived over large areas, surpassing the capabilities of traditional inventory or image analysis techniques. Urban areas pose particular challenges for remote sensing of tree structure, this is due to a heterogeneous and complex land cover as well as a wide range of potential tree structures. Here we presented methods and results for a new ALS Individual Tree Detection (ITD) method that is robust to a heterogeneous tree layer, allowing attribution of structure metrics from which AGB could be estimated. TLS provides highly accurate representations of tree structure and estimates of volume which were then used to develop local allometry. However, derivation of representative allometry for larger areas, including wood density values, continue to be a major source of uncertainty in estimating AGB, both in natural and urban forest. It should be noted that the ALS and TLS methods can be applied independently of each other, for example, literature allometry could be applied to the ITD method if TLS methods were unavailable. Owing to their proximity and inherent variabilities and idiosyncrasies in tree structure, urban forests provide an excellent testing ground for new methods and technologies to assess tree AGB.

## Abbreviations

AGB: above ground biomass
ALS: airborne laser scanning
*Ar*: projected crown area
*BIRCH*: balanced iterative reducing and clustering using hierarchies
C: carbon
CSM: canopy surface model
*dbh*: diameter at breast height
*DBSCAN*: density based spatial clustering and noise
*H*: maximum crown height
ITD: individual tree detection
LiDAR: light detection and ranging
MC: Monte Carlo
QSM: quantitative structure model
RMSE: root means square error
TLS: terrestrial laser scanning
UK EA: United Kingdom Environment Agency
*V*: tree volume
